# Hypochlorous-Acid-Generating Electrochemical Scaffold for Treatment of Wound Biofilms

**DOI:** 10.1038/s41598-019-38968-y

**Published:** 2019-02-25

**Authors:** Mia Mae Kiamco, Hannah M. Zmuda, Abdelrhman Mohamed, Douglas R. Call, Yash S. Raval, Robin Patel, Haluk Beyenal

**Affiliations:** 1 The Gene and Voiland School of Chemical Engineering and Bioengineering, Washington State University, Pullman, WA USA; 2 The Paul G. Allen School for Global Animal Health, Washington State University, Pullman, WA USA; 3 Divisions of Clinical Microbiology, Rochester, MN USA; 4 Divisions of Infectious Diseases, Mayo Clinic, Rochester, MN USA

## Abstract

Biofilm formation causes prolonged wound infections due to the dense biofilm structure, differential gene regulation to combat stress, and production of extracellular polymeric substances. *Acinetobacter baumannii*, *Staphylococcus aureus*, and *Pseudomonas aeruginosa* are three difficult-to-treat biofilm-forming bacteria frequently found in wound infections. This work describes a novel wound dressing in the form of an electrochemical scaffold (e-scaffold) that generates controlled, low concentrations of hypochlorous acid (HOCl) suitable for killing biofilm communities without substantially damaging host tissue. Production of HOCl near the e-scaffold surface was verified by measuring its concentration using needle-type microelectrodes. E-scaffolds producing 17, 10 and 7 mM HOCl completely eradicated *S. aureus*, *A. baumannii*, and *P. aeruginosa* biofilms after 3 hours, 2 hours, and 1 hour, respectively. Cytotoxicity and histopathological assessment showed no discernible harm to host tissues when e-scaffolds were applied to explant biofilms. The described strategy may provide a novel antibiotic-free strategy for treating persistent biofilm-associated infections, such as wound infections.

## Introduction

It is estimated that over 7.5 million people in the United States are affected by chronic wounds, with a projected financial burden on healthcare systems of $20 billion annually^1^. Individuals with chronic wounds often have an impaired inflammatory response and are unable to efficiently fight infectious microorganisms^1–3^ that acquire nutrients from injured tissue while resisting phagocytosis^3,4^. Moreover, 65% of all healthcare-associated infections have biofilms present in them according to a 2011 survey by the Centers for Disease Control and Prevention. Biofilms persist in chronic wounds and resist antibiotic treatments^5–7^.

Chronic wounds are associated with bacterial and/or fungal biofilm formation in the wound bed^2^. Biofilms are clustered microbial communities found on surfaces, embedded within a matrix known as extracellular polymeric substance (EPS) that protects the microbes from some antibiotics^8,9^. *Staphylococcus aureus*, *Pseudomonas aeruginosa*, and *Acinetobacter baumannii* are commonly associated with biofilm formation in wound infections^4,5,10–12^. Biofilms of *S. aureus* lead to a chronic infectious state in wounds by stimulating the production of pro-inflammatory cytokines and prolonging the inflammatory phase of the wound^1,13^. Public and healthcare concerns have arisen because of the increasing prevalence of bacteria resistant to antibiotics and antiseptics^10,14^. Thus, it is crucial to develop new treatment approaches to combat biofilm-infected wounds that do not involve antibiotics.

Electrical stimulation is broadly described as the act of passing an electric current across a wound bed via electrodes that are placed over the area of interest^15,16^. Electrical stimulation can increase tissue perfusion, decrease edema, promote angiogenesis, and direct cell migration in wound tissue, all of which can be important aspects of the wound repair process^17–19^. Moreover, such stimulation has demonstrated bactericidal effects against bacteria such as *P. aeruginosa*^20,21^ and *Escherichia coli*^22^.

The antibacterial mechanism of electrical stimulation is not well understood, which has in turn resulted in poor standardization of its applications^16,21,23–26^. A recent study demonstrated inhibition of *P. aeruginosa* by applying DC voltage (3.5 V); the authors ascribed this effect to electrochemical production of toxic compounds^27^, but this mechanism was not confirmed^23,25,27^. Moreover, electrical stimulation has varying effects that depend on how it is employed (e.g., DC *versus* AC, voltage and current settings, lengths of application time)^16,28^. For example, one study found that the application of electric current in a copper mesh electrode at a current density of 32 μA/cm^2^ for 2 hours, 3 times daily resulted in elimination of *P. aeruginosa* from infected skin ulcers^21^ while a similar study required the application of electrical current on the same material at a current density of 52 μA/cm^2^ for 72 hours to eradicate *P. aeruginosa* in an infected wound model^20^. Exploring electrochemical processes occurring on electrode surfaces should make it possible to standardize these applications.

Our previous publications^28,29^ showed that hydrogen peroxide (H_2_O_2_) can be produced at controlled concentrations near the surface of an electrochemical scaffold by keeping the scaffold at a suitable fixed potential. Our H_2_O_2_-producing electrochemical scaffold (e-scaffold) eliminated large (4-log) microbial communities in biofilms. Moreover, the H_2_O_2_-producing e-scaffold successfully eradicated *A. baumanii* biofilms when combined with a hyperosmotic agent, maltodextrin, after 24 hours of treatment. For the current work, we developed an e-scaffold that produces hypochlorous acid (HOCl) near the electrode surface. HOCl acts faster to eradicate pathogenic bacteria and has lower cytotoxicity towards mammalian cells than sodium hypochlorite (NaOCl) or H_2_O_2_^30^. HOCl eradicates bacteria by inhibiting bacterial growth, cell division, and protein synthesis; oxidizing sulfhydryl enzymes and amino acids; decreasing adenosine triphosphate production; breaking DNA; and depressing DNA synthesis^22,31,32^. HOCl is already used as a wound cleansing agent^33^, and published work demonstrates that HOCl acts as an antimicrobial, anti-biofilm agent that also promotes wound healing^30,34–37^. Sakarya *et al*. showed that a stabilized HOCl solution decreased cell numbers in biofilms and had favorable effects on fibroblast and keratinocyte migration *in vitro* compared to povidone iodine^34^. Previous studies showed that HOCl solution is not painful to patients, eliminates wound odor^38^, and is effective in cleansing, debriding, and promoting wound healing^39^. HOCl is considered an excellent adjunct therapy for infection treatment as well as a safe and effective wound cleansing agent^40^.

The goal of this study was to develop a technology that would allow the sustained delivery of controlled concentrations of HOCl to a biofilm wound bed. To the authors’ knowledge, this is the first report of a potentiostatically controlled wound dressing delivering continuous controlled concentrations of HOCl to eradicate wound biofilms. The e-scaffold is a device that oxidizes chloride ions (Cl^−^) from phosphate-buffered saline (PBS) or physiological chloride ions (Cl^−^) in blood and wound fluid to chlorine (Cl_2_) (Equation 1^41^); the Cl_2_ then reacts with water to produce HOCl (Equation 2). Low concentrations (below a CT_50_ of 286 μM^30^) of HOCl can be antimicrobial without causing cytotoxicity to host tissue^40^. Our hypothesis is that HOCl generated electrochemically by the e-scaffold can eliminate biofilms without damaging host tissue.

1 $$2{{\rm{Cl}}}_{(\mathrm{aq})}^{-}\iff {{\rm{Cl}}}_{2(g)}+2{{\rm{e}}}^{-}\,\,\,\,\,{{\rm{E}}}_{{\rm{o}}}=+\,1.138\,{{\rm{V}}}_{\mathrm{Ag}/\mathrm{AgCl}}$$

2 $${{\rm{Cl}}}_{2(g)}+{{\rm{H}}}_{2}{\rm{O}}\iff {{\rm{Cl}}}_{(\mathrm{aq})}^{-}+{{\rm{HOCl}}}_{({\rm{aq}})}+{{\rm{H}}}_{(\mathrm{aq})}^{+}$$

To verify HOCl generation, custom-made microelectrodes were employed to measure HOCl concentrations near the surface of the e-scaffold. *S. aureus*, *A. baumannii* and *P. aeruginosa* biofilms were subjected to HOCl e-scaffold treatment for various lengths of time. The addition of exogenous HOCl for biofilm treatment was also compared to e-scaffold-generated HOCl. Finally, uninfected *ex vivo* porcine explants were subjected to HOCl- producing e-scaffolds for a preliminary assessment of cytotoxicity, and HOCl e-scaffold efficacy was tested against infected porcine explants. The e-scaffold produces a continuous supply of HOCl at a low concentration, a process that is very different from simply applying HOCl directly to a wound. By using a three-electrode system, it is possible to control reactions on the e-scaffold and quantify the amount of generated HOCl. This allows us to control the concentration of HOCl, which is a significant difference from previous studies.

## Results and Discussion

### HOCl is generated on the electrochemical scaffold surface

**Figure 1:**
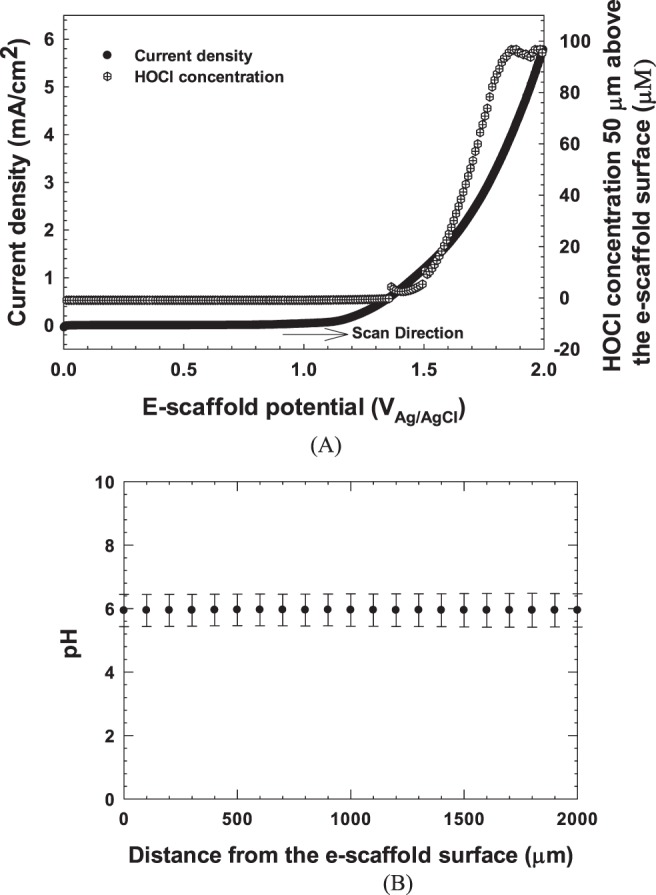
HOCl is generated on the electrochemical scaffold surface. (**A**) Change in HOCl relative to e-scaffold potential. The microelectrode tip was located ~50 µm above the e-scaffold surface, and the HOCl concentration was monitored while the electrode-scaffold potential was varied from 0 V_Ag/AgCl_ to +2.0 V_Ag/AgCl_ at a 0.010 V/s scan rate. The onset potential of HOCl generation occurred at ~1.38 V_Ag/AgCl_. The discontinuity of the concentration profile (top panel) is a result of the automatic current range adjustment in the Gamry© Interface 1000E^TM^ potentiostat used to measure current. As HOCl concentration increases, the current measured at the microelectrode increases, pushing the instrument to the next current range. (**B**) The pH variation with distance from the e-scaffold surface while HOCl was produced at +1.5 V_Ag/AgCl_. Error bars represent standard deviation from the average of 3 separate measurements.

Application of a potential above +1.138 V_Ag/AgCl_ generated HOCl electrochemically at ~50 µm from the surface of the e-scaffold (Fig. 1A). Approximately 5 µM HOCl was produced at a potential of +1.5 V_Ag/AgCl_ (Fig. 1A). This value is below the reported toxic concentration of HOCl^30^, and we therefore used +1.5 V_Ag/AgCl_ for the remaining experiments. The production of HOCl did not cause variation in pH, as the pH measured near the surface of the e-scaffold was similar to the pH measured 2,000 µm above the surface (Fig. 1B). This is important because HOCl exists in equilibrium with OCl^−^ and Cl_2_ gas. At pH 7.5 (pKa of HOCl), HOCl is present in equal amounts with OCl^−^, while Cl_2_ exists prominently at lower pH^42^. With the pH of the solution between 3.5 and 6.5, HOCl will be the predominant species relative to either OCl^−^ or Cl_2_. Operating at pH below 6.5 also has beneficial impacts on wound healing. Chronic wounds have a pH range of 7.15–8.9^43^, but delivery of oxygen from red blood cells is more efficient when the pH is <7.4^44^. Moreover, reports show that lowering the wound pH using Manuka honey can lead to a decreased wound size^45^. Furthermore, studies show that low pH promotes epithelialization of wounds^43,46–48^ and can inhibit bacterial growth and reduce activities of proteases that are detrimental to wound healing^49^. Our e-scaffold does not change the pH in the wound bed and thus does not cause undesired effects.

### *In vitro* biofilms were eradicated by the HOCl- generating electrochemical scaffold

**Figure 2:**
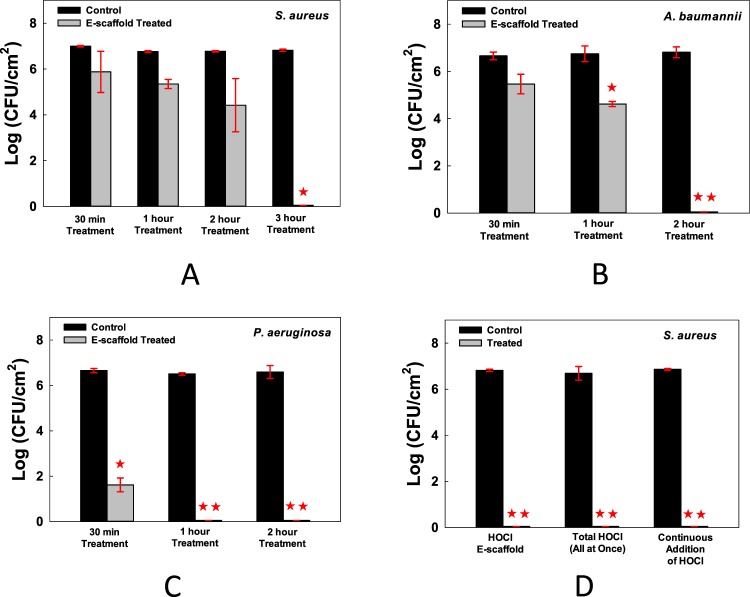
*In vitro* biofilms were eradicated by the HOCl- generating electrochemical scaffold. The HOCl- generating e-scaffold completely eradicated viable cells of (**A**) *S. aureus* biofilms after 3 hours, (**B**) *A. baumannii* biofilms after 2 hours, and (**C**) *P. aeruginosa* biofilms after 1 hour. Data are means, and errors are standard deviations of 3 biological replicates. (**D**) *S. aureus* biofilm cell viability after treatment with HOCl e-scaffold, final (17 mM) HOCl concentration, and continuous HOCl addition for 3 hours. Data are represented as mean ± SD; n = 3. Asterisks indicate statistically significant differences from the control biofilm (*P* < 0.001, and 0.001 < *P* < 0.05).

All viable *S. aureus* cells were eradicated after 3 hours of treatment with the HOCl e-scaffold (Fig. 2A), all viable *A. baumannii* cells were eradicated after 2 hours of treatment (Fig. 2B), and all viable *P. aeruginosa* cells were eradicated after 1 hour of exposure (Fig. 2C).

### E-scaffold-generated HOCl and exogenously added HOCl show similar efficacy

To determine the mechanism by which HOCl kills biofilm cells, we investigated two possibilities: (1) pH change to a highly alkaline state eradicates biofilms, and (2) HOCl is the dominant reactive chlorine species produced during the e-scaffold-mediated oxidation process. Changes in pH are unlikely, as shown in Fig. 1B (the pH stayed at ~6, which is within the range, 6.5–3.5, where HOCl predominates). To determine whether HOCl is the sole effector against biofilms, we compared the efficacy of the e-scaffold to that of HOCl added exogenously either as a single dose or continuously, which mimics the e-scaffold mode of operation. We estimated that a 3-hour exposure to the e-scaffold produced a total of 0.12 mmoles (=17 mM) of HOCl. When we treated *S. aureus* biofilms for 3 hours with the scaffold or by introducing 0.12 mmoles of HOCl exogenously (at one time or by continuously adding 150.43 mM HOCl solution at a rate of 5.25 μL/minute over a 3-hour period), the biofilms were eradicated (Fig. 2D), consistent with HOCl being the dominant compound killing the biofilm cells. Adding the total amount of HOCl exogenously at one time eradicated biofilm as well as the HOCl e-scaffold; the drawback of this approach is that the initial high concentration exceeds the reported cytotoxicity limit (286–477 μM)^30^. Continuously adding HOCl presents challenges including how to control the concentration near the biofilm, whereas such control is an inherent property of the e-scaffold.

**Figure 3:**
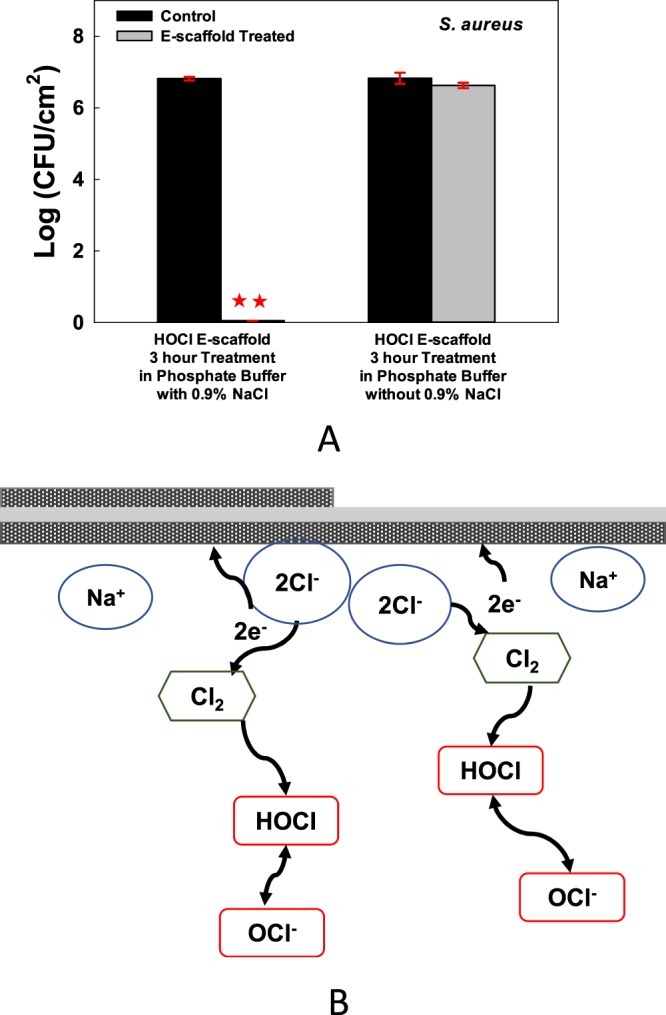
HOCl generation is the dominant mechanism of biofilm eradication. (**A**) No significant reduction was observed in biofilms treated with e-scaffold in phosphate buffer only (no Cl^−^). Data are represented as mean ± SD; n = 3. Polarization at +1.5 V_Ag/AgCl_ (current densities of 0.33 ± 0.09 mA/cm^2^ and 0.13 ± 0.02 mA/cm^2^ for PBS and PB, respectively). The asterisks indicate statistically significant differences from the control biofilm (*P* < 0.001). (**B**) Proposed mechanism of action of HOCl e-scaffold. Red boxes indicate active anti-biofilm species.

To test whether electrochemically generated HOCl is the dominant mechanism for biofilm removal, we removed the Cl^−^ from the medium, which prevents the production of HOCl when the e-scaffold is operated at suitable potentials. To do this we used custom-prepared phosphate buffer solution with 32 mM dipotassium phosphate (K_2_HPO_4_) and 18 mM monopotassium phosphate (KH_2_PO_4_). No KCl was added to the solution, and the pH was adjusted using 1 M NaOH or 1 M HNO_3_, when needed. When phosphate buffer solution was used, the e-scaffold killing mechanism was neutralized (Fig. 3A). This shows that the presence of Cl^−^ ions is key in the electrochemistry of the HOCl e-scaffold. Although this does not mean that HOCl is the only mechanism involved, we can conclude that HOCl is the dominant species present in the solution and is mostly responsible for *in vitro* anti-biofilm activity. At pH ranging between 6.3 and 6.7, an average of 90% of Cl^−^ oxidation product is HOCl, with the remainder OCl^−^ (<10%)^30^, and it is likely that both species contribute to the antibacterial effects of the e-scaffold (Fig. 3B).

We should note that the data in Fig. 3A also show that the use of an Ag/AgCl electrode did not eradicate biofilms, because a polarized electrode was used when Cl^−^ was removed. Moreover, we found that the e-scaffold and control showed nearly identical bacterial counts after 24 hours when the electrode was not polarized (Fig. SI 2). This control experiment demonstrates that none of the e-scaffold components (including the Ag/AgCl reference electrode) had biocidal effects.

### Electrochemically produced HOCl does not damage host tissue and eradicates *ex vivo* biofilms

**Figure 4:**
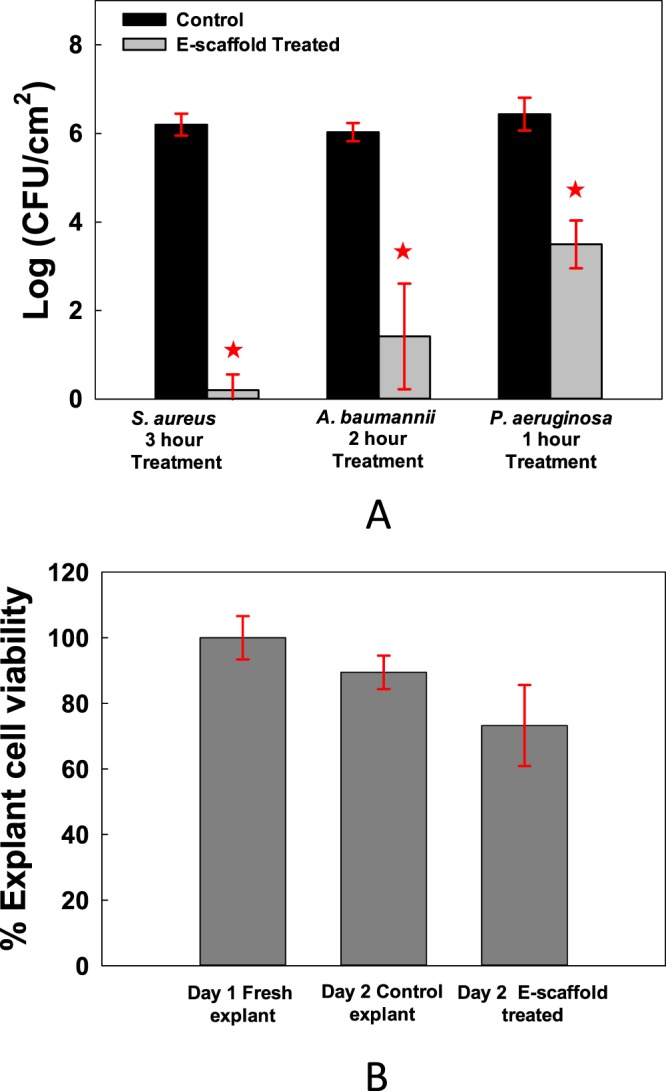
Electrochemically produced HOCl eradicates biofilms on explants without reducing cell viability. (**A**) The HOCl e-scaffold decreases the number of viable cells of *S. aureus*, *A. baumannii*, and *P. aeruginosa* biofilms on infected porcine explants. (**B**) The HOCl e-scaffold does not significantly decrease explant cell viability. Data are represented as mean ± SD; n = 3. Asterisks indicate statistically significant differences from the control biofilm (*P* < 0.001, and 0.001 <*P* < 0.05).

**Figure 5:**
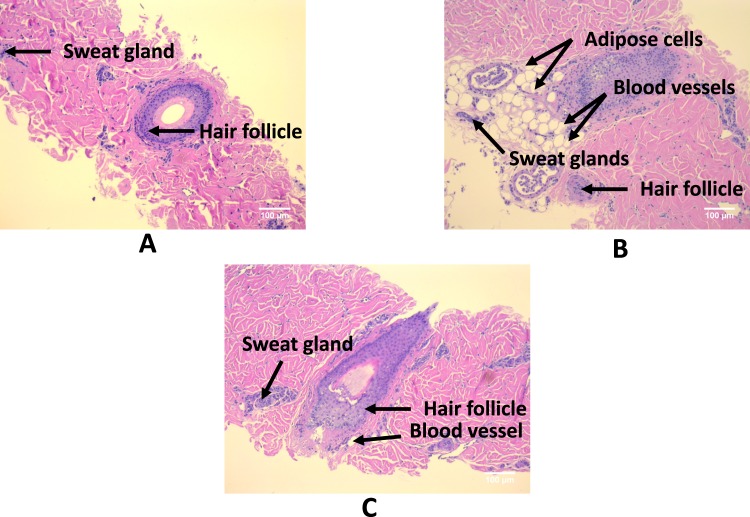
Electrochemically produced HOCl does not damage host tissue: Cross-sectional micrographs (100×) showing hair shafts/hair follicles and dermis. The epidermal layer was removed for the experiment). No gross pathology was evident from any of the images. (**A**) Fresh explant showing adipose cells, sweat glands and blood vessels. Isolated hair follicles have epithelium with prominent clear cytoplasm. (**B**) Micrograph of 1-day-old untreated explant. (**C**) Micrograph image of 1-day-old e-scaffold-treated explant (the e-scaffold was applied and polarized at +1.5 V_Ag/AgCl_ for 3 hours).

The efficacy of the HOCl e-scaffold was tested on biofilms grown on porcine dermal explants. After 3 hours of exposure to the HOCl e-scaffold, *S. aureus* biofilm cell counts dropped by ~6 logs (Fig. 4A). Moreover, the HOCl e-scaffold reduced viable *A. baumannii* and *P. aeruginosa* biofilm cells by ~5 log and ~3 log after 2 and 1 hours of treatment, respectively. The HOCl e-scaffold was more effective than a previously described H_2_O_2_ e-scaffold^28^ (~5 log-reduction of viable cells compared to ~3 log-reduction by the H_2_O_2_ e-scaffold) both in terms of log reduction and time required. Cytotoxicity studies showed that HOCl- exposed porcine dermal explants did not decline in viability with e-scaffold treatment (Fig. 4B). Moreover, blinded histopathological assessment did not show significant differences between the untreated and treated porcine dermal explants (Fig. 5). These results suggest that a 3-hour exposure to a HOCl e-scaffold does not damage host tissue.

Biofilms contain extracellular polymeric substance (EPS), which restricts the diffusion of molecules and can bind to some antimicrobials. For example, the negatively charged EPS protects cells by preventing positively charged aminoglycoside antibiotics from permeating into the biofilm, possibly through electrostatic interactions^50,51^. Davenport *et al*.^52^ demonstrated that *A. baumannii* is protected against tobramycin regardless of which species of pathogen produces the EPS. Nevertheless, previous literature showed that the reduction of antibiotic penetration varies depending on the bacteria and antibiotic used^53^. Biofilms also restrict penetration by biocides other than antibiotics, such as H_2_O_2_^54,55^ and HOCl^56^. It is our contention that by continuously generating HOCl at low concentrations, this restriction can be mitigated. In addition, HOCl is 80–100 times more potent as a germicide than NaOCl because HOCl is neutral and penetrates bacteria more readily than the charged OCl^−^^57^. From the cellular perspective, Gram-positive bacteria have a thicker peptidoglycan layer than Gram-negative bacteria; peptidoglycan is a covalently linked polymer matrix that is composed of peptide-linked β-(1–4)-*N*-acetyl hexosamine^58^. This added thickness makes Gram-positive bacteria more resistant to mechanical and chemical stress^59^, which may explain the longer time required to kill *S. aureus* biofilm cells (Fig. 2).

The current study used a three-electrode system, whereas earlier work by Sandvik *et al*. (2013) employed a current-controlled, two-electrode system with which the presence of free chlorine was demonstrated^26^. The authors concluded that electric current killed *S. epidermidis*. We used a three-electrode system to control the potential of the working electrode to generate HOCl, which allowed the development of a system that is independent of electrode size and system geometry^60^.

**Figure 6:**
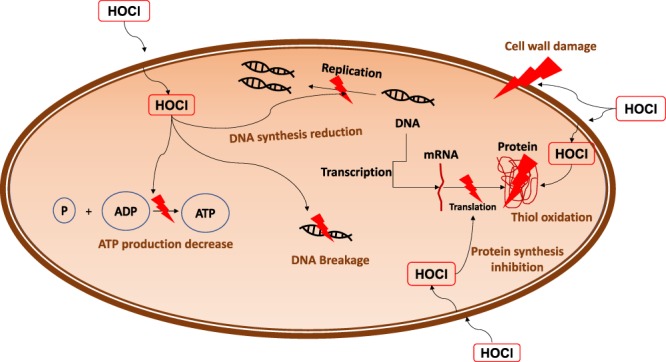
Proposed mechanism of action: Schematic of HOCl action against bacterial cells. Uncharged HOCl readily penetrates the cell wall and inhibits DNA synthesis, protein synthesis (oxidation of thiol- containing proteins and enzymes), and bacterial growth (through depressing DNA replication and inhibiting cell wall synthesis). HOCl also affects bacterial metabolism through decreasing ATP production.

HOCl is a naturally occurring compound that is one of several oxidative species (including O^−^_2_ and H_2_O_2_) that are produced by neutrophils and macrophages^61,62^. Inside bacterial cells, HOCl decreases ATP production in *E. coli*, as well as in *Pseudomonas* and *Staphylococcus* species^63^. HOCl eradicates cells by inhibiting bacterial growth, cell division, and protein synthesis; oxidizing sulfhydryl enzymes and amino acids; decreasing adenosine triphosphate production; breaking DNA; and depressing DNA synthesis^22,31,32^. Figure 6 illustrates the multi-mechanism action of HOCl.

Some current literature supports the claim that electrical stimulation accelerates wound healing through galvanotaxis^64,65^ and provides antimicrobial effects^20,21,66^. Methods of application include dressings, electrode placement, and practitioner-assisted control^65,67–69^. Bioelectric dressings such as Procellera® and Posifect RD® DC are emerging as a useful method for delivering electrical stimulation. Both dressings are focused on accelerated wound healing^67,70,71^. Electrical stimulation studies vary with respect to which modality works best against infection; they employ various ranges of DC voltages, current settings, polarity of the electrodes placed on wounds, lengths of application and other variables^16,28^. Generally, researchers hypothesize that electrical current and electrical fields are responsible for antimicrobial effects^20,21,66,72^ but acknowledge that the production of toxic compounds may also play a role. Our work demonstrates a specific mode of action in which HOCl is produced near the surface of a specifically tuned e-scaffold and eradicates bacterial biofilms.

The presented work establishes a foundation for an alternative antibiotic-free strategy for treating persistent biofilm infections on wound surfaces through the controlled local generation of HOCl near the infected wound surface.

## Methods

### Electrochemical scaffold assembly

**Figure 7:**
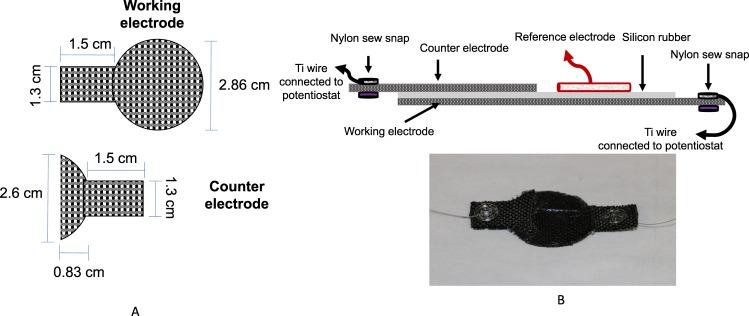
(**A**) Dimensions of the e-scaffold components. (**B**) Detailed description of the components (not drawn to scale) and a photograph of the electrochemical scaffold assembly.

An e-scaffold with a 2.86-mm diameter was used for *in vitro* experiments. Carbon fabric (Zoltek Companies Inc., St. Louis, MO, Panex 30 PW-06) was used as the electrode material because of its flexibility and electrochemical performance. The working electrode was cut into a circular shape (diameter of 2.86 cm) with a 1.3 × 1.5 cm rectangular tail for attaching the wire (Fig. 7A top). The counter electrode was cut to a short-half-circle shape with a diameter of 2.6 cm with a same-sized rectangular tail (Fig. 7A). This was done because we wanted to use the extra space on the ventral side of the working electrode as a pseudo-reference electrode (for future research projects). A thin layer of silicone rubber was placed between the working and counter electrodes to prevent electrical contact and to improve e-scaffold rigidity. Before assembly, the carbon fabric was treated in 1 M HCl overnight and washed with deionized water. Nylon sew snaps (Dritz, Spartanburg, SC, item #85) were used to connect titanium wires (TEMCo, Amazon.com, catalog #RW0524) to the fabric by looping the wire into the snap before attaching it to the fabric. Silicone adhesive was used to affix the working and counter electrodes, which were kept out of direct contact with one another (Fig. 7B). A custom-made Ag/AgCl reference electrode was constructed according to published protocols^73^. We confirmed that the reference electrode by itself did not eradicate biofilms. New e-scaffolds were used for each experiment. For quality control, the ohmic resistance between the titanium wire and the carbon fabric was measured using a Fluke 87 V multimeter (Fluke, Everr, WA). E-scaffolds with a resistance higher than 5 Ω were discarded. Ti wire was used to connect carbon fabric to the potentiostat; ~0.5 cm of the Ti wire was exposed to the solution. We used Ti wires with a 150-μm diameter, and the total surface area of the Ti wires (~5 × 10^−4^ cm^2^) was significantly less (by a factor of >10,000) than the total surface area of the working electrode (~10.9 cm^2^). The resistance between the Ti wire and the carbon fabric was measured before and after the experiments, and we detected no change. For our e-scaffolds, the working electrode is equivalent to an anode and the counter electrode is equivalent to a cathode.

### HOCl concentration and pH measurements

The HOCl microelectrode was constructed similarly to an H_2_O_2_ microelectrode following previously described protocols^73^. However, to make the HOCl microelectrodes work correctly in the electrical field, we built an integrated reference electrode. This allowed us to place the tip and reference electrodes very close to each other and move them together without changing the distance between them. The construction processes of H_2_O_2_ and HOCl microelectrodes are identical; the only difference is the polarization potential used for detecting HOCl. Details of the construction process are published elsewhere^74^. The microelectrodes had tip diameters of <20 µm. HOCl microelectrodes were polarized to +0.35 V_Ag/AgCl_ and calibrated using a sodium hypochlorite (NaOCl) solution diluted in PBS (initial pH = 6.3) with a concentration ranging from 0 to 600 µM. The pH of the calibration solutions did not vary significantly with NaOCl concentration (~0.1 pH unit). An example calibration curve is shown in Fig. SI 2. The microelectrode response was linear from 0 to 157 µM, with a detection limit of 0.25 µM (S/N = 2) and a response time of less than three seconds. Custom-made Ag/AgCl reference electrodes were used for HOCl measurements^73^. Two independent Gamry© Interface 1000E^TM^ potentiostats (Gamry© Instruments, Warminster, PA) were used to operate the HOCl microelectrode and the e-scaffold. The microelectrode was initially placed on top of the working electrode side of the e-scaffold, which was located using a Zeiss Stemi 2000 stereomicroscope (Carl Zeiss© Microscopy). The e-scaffold was placed in a 6-well plate containing 7 mL of PBS with an initial pH of 6.3. A stationary profile of the HOCl concentration near the surface (~50 µm) of the e-scaffold was measured. A stationary profile was obtained by placing the microelectrode tip ~50 µm above the e-scaffold surface (to measure HOCl concentration) while changing the potential of the working electrode from 0 V_Ag/AgCl_ to 2 V_Ag/AgCl_ with a scan rate of 0.010 V/s. During the stationary profile measurement, the microelectrode tip was not moved but the electrode potential changed. This allowed us to determine the onset potential of HOCl generation (Fig. 1A). The electrical current response from the HOCl microelectrode during the scan was recorded. This response was later used to calculate the concentration of HOCl. A computer-controlled stepper motor (Physik Instrumente©, PI M-230.10 S, part no. M23010SX) linked to custom LabVIEW software (National Instruments, Austin, TX, USA) was used to manipulate microelectrode movements and control the location of the tip. The vertical range of the motorized actuator was 8 mm with a 40-nm resolution. After the microprofiling system was set up, the HOCl microelectrode was placed within the micromanipulator arm. A stereomicroscope (Zeiss Stemi 2000) was used to locate the tip of the microelectrode held by the micromanipulator. Using the coarse knobs on the micromanipulator, the microelectrode tip was moved above the scaffold, lowered, and positioned to near the e-scaffold surface. The stepper motor was then used to move the microelectrode with a step size of 5 μm until the tip touched the e-scaffold surface, which is used as a reference point for distance. The microelectrode was then moved to ~50 µm above the e-scaffold surface to measure the stationary HOCl profiles.

The pH microelectrode was constructed following previously described protocols^73^. The microelectrodes had tip diameters of <20 µm and were constructed with liquid ion exchange (LIX, Sigma-Aldrich, 95297) membrane tips^73^. Custom-made Ag/AgCl reference electrodes with an agar salt bridge at the tip were used for pH measurements. The pH microelectrodes were calibrated in buffer solutions (pH = 4, 7, and 10, Cole-Palmer 910104, 910107, and 910110). The pH measurements were performed while the e-scaffold was polarized at +1.5 V_Ag/AgCl_. The pH profiles were recorded when the current of the polarized e-scaffold reached a steady state (~22 minutes of polarization). The pH microelectrode was moved using the stepper motor in the z-direction at a displacement rate of 100 µm every 10 seconds. The pH sensor output was recorded using a Kiethley© 6537 Electrometer. The pH measurements were performed in an experimental setup and under conditions identical to those of the HOCl microelectrode measurements. The bulk pH before and after the measurements varied between 6.3 and 6.4.

### Bacterial strains, media and culture preparation

Pure cultures of *S. aureus* (ATCC BAA-1747), *A. baumannii* (ATCC #BAA-1605), and *P. aeruginosa* PAO1 were used in this study. All organisms were plated on trypticase soy agar (TSA) (Fisher Scientific, DF0369-17-6). Trypticase soy broth (TSB) (Fisher Scientific, DF0370-17-3) was used to culture *S. aureus* and *A. baumannii*, and Lennox L Broth (LB) (ThermoFisher, 12780–052) was used to culture *P. aeruginosa*. To prepare the inoculum, 5 mL of medium and 1 colony of bacteria were added to a 50-mL polypropylene tube and incubated at 37 °C on a shaker table (75 rpm, overnight growth, stationary incubator).

### Biofilm preparation

Overnight cultures (18 hours of growth in a 37 °C incubator, shaking at ~90 rpm) of *S. aureus*, *A. baumannii*, and *P. aeruginosa* were diluted to an OD_600_ of 0.5. One milliliter of a diluted culture was dispensed to each well in a 6-well plate (VWR Intnl Inc, 29442–042) and left for 1 hour for cell attachment. The wells were then washed twice with 1 mL of sterile TSB. TSB (2 mL) was added, and the plates were incubated for 24 hours at room temperature. The medium was replaced, and incubation was continued for 48 hours total. LB was used for *P. aeruginosa* biofilm growth instead of TSB.

### Electrochemical scaffold treatment of *in vitro* biofilms

**Figure 8:**
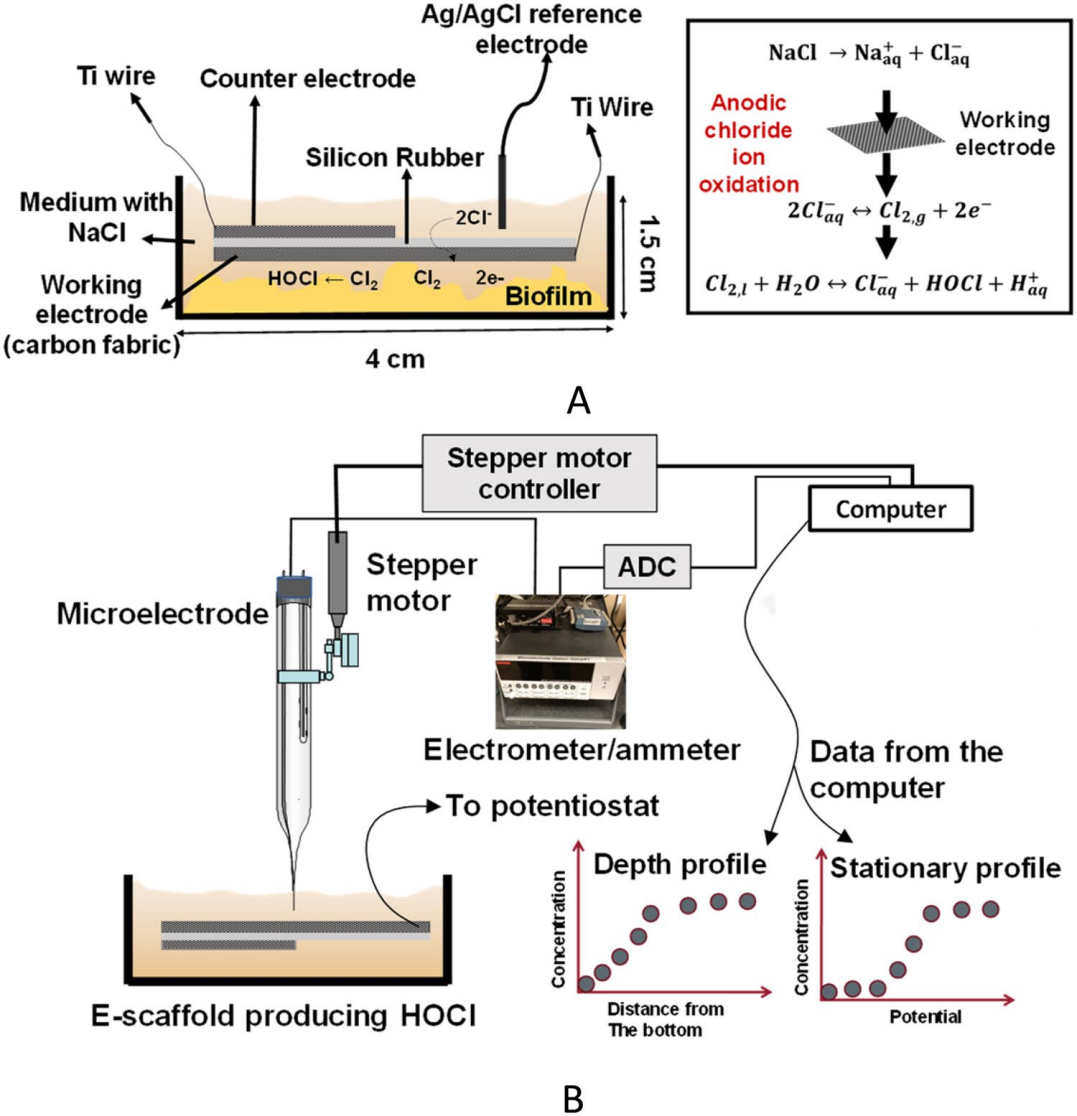
(**A**) Schematic of experimental setup for treatment of *in vitro* biofilm with HOCl that is produced and the e-scaffold reaction. The e-scaffold was placed on top of a biofilm grown in one of the 6-well plates. Chloride ions supplied by the 0.9% PBS are oxidized to chlorine at the working electrode when a positive potential is applied to the working electrode. Chlorine dissociates into the solution to form HOCl driven by the pH of the solution. (**B**) Experimental setup used to measure pH profiles. For HOCl profiles, a Gamry© Interface 1000E^TM^ was used instead of the electrometer/ammeter and ADC. The figures are not drawn to scale.

After 48 hours of biofilm growth, spent medium on the biofilms was replaced with 7 mL of sterilized PBS solution (32 mM dipotassium phosphate (K_2_HPO_4_), 18 mM monopotassium phosphate (KH_2_PO_4_), and 0.9% w/v sodium chloride (NaCl), pH 6.3-6.35). The PBS was titrated with 1 M HNO_3_ or 1 M NaOH in the event that the pH was above or below the desired pH. Next, the e-scaffold was placed directly on top of the biofilm and then polarized at +1.5 V_Ag/AgCl_ for the selected treatment time. While we were unable to measure the exact distance between the e-scaffold and the biofilm, this e-scaffold is made of flexible fabric electrodes that conform to the surface below them. The potential at the working electrode was ~ −1.6 V_Ag/AgCl_, which is well below the compliance voltage of the potentiostat, indicating that the counter electrode did not limit the current. The surface areas of the working and counter electrodes exposed to the PBS were ~6.42 cm^2^ and ~2.5 cm^2^, respectively. The e-scaffold was conditioned twice (in PBS only and in PBS on biofilm) prior to being polarized by running cyclic voltammetry (0.100 V/s and 0.010 V/s scan rates, 3 cycles, scan from 0 V_Ag/AgCl_ to 2 V_Ag/AgCl_). To test whether conditioning affects biofilms prior to polarization, a control experiment with e-scaffold (but no polarization) was performed. When we conditioned the e-scaffold in PBS, there was no detectable killing of the biofilm (Fig. SI 1). Untreated biofilms were used as a control (this control did not include an e-scaffold or a reference electrode). A control experiment without the presence of chloride ions was also performed to determine whether the dominant species produced with the e-scaffold surface is HOCl. The e-scaffold and Ag/AgCl reference electrode were positioned similarly to their positioning in the setup shown in Fig. 8A and polarized for 3 hours (3 hours was chosen for this control because it is the longest treatment duration used in our experiments). This control also determined whether the Ag/AgCl reference electrode itself has any killing effect on the biofilm. After treatment, biofilms were scraped from the surface of the 6-well plate, vortexed to homogenize the biofilms, and washed with 1 mL of PBS three times (with centrifugation in between). The biofilms were harvested after the duration of the treatment times (3 hours, 2 hours, 1 hour, and 30 mins). Viable cell counts were determined using the drop plate method^75^. Figure 8 shows the experimental setup for testing the e-scaffold on *in vitro* biofilms and the scheme of electrochemical reactions.

### Exogenous addition of HOCl

*S. aureus* was chosen for this test because its cell membrane is more difficult to penetrate than those of the other bacteria used in this study. *S. aureus* biofilms were prepared in a 6-well plate using the same protocol described for *in vitro* biofilm treatment. Exogenous HOCl was added to the biofilm well to reach a final concentration equivalent to the HOCl concentration produced by the e-scaffold. To calculate the theoretical total amount of HOCl produced on the e-scaffold after 3 hours, we integrated the current passed over the treatment period to calculate the charge (C) and divided by the Faraday constant (96,487 C/mol). This gives the moles of electrons exchanged with the solution. We assumed that the oxidation reaction of Cl^−^ ions dominated the measured anodic current according to Equation 1 (assuming 100% Faradaic efficiency). We also assumed that the Cl_2_ gas generated in the medium was completely converted to free reactive chlorine compounds according to Equation 2. The final concentration of HOCl produced was calculated from the HOCl equivalent of Cl^−^ oxidation divided by the solution volume. Overall, the equivalent of 0.12 mmoles (17 mM) of HOCl in the well was generated by the e-scaffold after 3 hours. This translates to a ~6 mM increase/hour for the 3-hour treatment. Thus, the volumetric flow rate, 314.76 µL/hour, was used for the continuous addition of HOCl to *S. aureus* biofilms. From the stock solution of NaOCl (10–15% w/v available chlorine, 1.4 M–2.1 M NaOCl, Sigma Aldrich, 424044), a concentration of 150.43 mM was prepared and aliquoted using a 3-mL syringe (BD, Fisher Scientific, 14-823-436). The spent medium on the *S. aureus* biofilm was replaced with 7 mL of PBS. Then, using a Legato 270 P syringe pump (kd Scientific, 78–8272), HOCl solution was pumped into the biofilm at a volumetric flow rate of 314.76 µL/hour.

From the remaining solution of 150.43 mM, a concentration of 17 mM was prepared in an 8-mL total volume of PBS. About 7 mL of PBS was added to the control biofilm, while 7 mL of 17 mM HOCl was added to *S. aureus* biofilms and incubated for 3 hours. After the exogenous HOCl treatments, biofilms were harvested by scraping the 6-well plate surface. The biofilms were homogenized and washed 3 times with 1 mL of PBS (with centrifugation in between to collect cell pellets). Cell counts were performed using the drop plate method^75^.

### Explant preparation, cytotoxicity determination, and histopathology

We followed previously established protocols^76,77^. Ear samples were obtained from a local abattoir (C&L Lockers, Moscow, ID) at the time that private client healthy pigs were butchered. Fresh ears were immediately cooled to 4 °C during transport and kept for less than an hour at this temperature before being processed in the laboratory. The tissues were cleaned with 70% ethanol, and the hair was removed using an electric clipper. The epidermis layer was excised with a Padgett’s dermatome and discarded. Then the dermis layer was excised and placed in a Petri dish containing 20 mL of serum-free Dulbecco’s modified Eagle’s medium (DMEM) (Thermo Scientific, 11995040) with no supplement. The excised dermis layer was sectioned at a thickness of approximately 500 μm, using a Padgett’s dermatome, and punched into 12-mm-diameter discs, excluding layers with visible skin abnormalities (scratches, erosion or scars). Next, the dermis layer punches were used with the dermal side down to seed polycarbonate transwell inserts (Greiner Bio-One North America, Inc., 657641) with a 0.4-μm pore size membrane separating each explant from the outer well prefilled with 2 mL of cell nutrient medium. The nutrient medium consisted of DMEM supplemented with ampicillin (50 μg/mL) and amphotericin B (0.4 μg/mL).

### Cytotoxicity

For cytotoxicity tests, uninfected explants (after 24-hour incubation at 37 °C, 95% humidity, and 5% CO_2_) were exposed to the HOCl e-scaffold for 3 hours, and so were 2 controls: (1) explants processed on the day of the pig ear extraction (day 1 fresh explant) and (2) explants that were not exposed to the e-scaffold (day 2 control explant). After application of the e-scaffold (12 mm diameter working electrode), porcine explant cell viability was quantified using the PrestoBlue cell viability reagent (Thermo Fisher, A-13262) per standard protocols (Life Technologies). Briefly, explants were incubated in 300 μL of 10% PrestoBlue (in DMEM) for 3 hours at 37 °C. Absorbance of the medium was then measured at 570 nm and 600 nm. The percent reduction of PrestoBlue was calculated from this absorbance and the molar extinction coefficient of oxidized and reduced PrestoBlue. A normalized viability score of 100% was assigned to the explant showing the highest percent reduction of PrestoBlue for this control.

### Histopathology

Explants exposed to HOCl e-scaffold and PBS only (control) were fixed in 10% formalin for histopathological analysis. For this experiment, the explants subjected to histopathological analysis were independent from the explants used for cytotoxicity testing. The resulting light micrographs were subjected to treatment-blind evaluation by a board-certified anatomic and clinical pathologist. For each case, we used independent explants (3 biological replicates for each treatment). We imaged each sample from each replicate from several locations on the slide (total of 4–6 images), and all the observed images were consistent (only select images are shown here).

### Biofilm-infected explants

Explants were processed as described above. Overnight cultures of *S. aureus*, *A. baumannii*, and *P. aeruginosa* were diluted to an OD_600_ of 0.5. Explants were infected with 5 μL of diluted bacteria (~1 × 10^9^ CF/mL). After 4 days of biofilm growth (inside an incubator at 37 °C, 95% humidity, and 5% CO_2_), explants were subjected to e-scaffold treatment as described above for *in vitro* biofilms (the e-scaffold was overlaid on the explant, which was placed on a transwell insert). Four milliliters of PBS (pH = 6.3) were added into the transwell insert as electrolyte. Treatment times were 3 hours for *S. aureus*, 2 hours for *A. baumannii*, and 1 hour for *P. aeruginosa*. Biofilms from the explants were collected by scraping and vortexing the explant in PBS. Then the biofilm cells were washed 3 times in 1 mL of PBS. Finally, viable cells were evaluated using the drop plate method^75^.

### Data analysis

All experiments were performed at least in triplicate. *In vitro* and *ex vivo* CFU data were analyzed using a t-test to identify statistically significant differences. A one-way ANOVA was used with a Tukey test to identify any significant differences in the explant viability test. We considered *P* ≤ 0.05 to be the threshold for statistical significance. Calculations and statistical analyses were performed using Sigma Plot© (versions 12.0 and 12.5).

## Supplementary information

Supplementary Information

## Data Availability

The data supporting the findings in this work are available from the corresponding authors upon request.
