# Supplementary material for: Hypochlorous-Acid-Generating Electrochemical Scaffold for Treatment of Wound Biofilms
Source: Sci Rep. 2019 Feb 25;9:2683. doi: 10.1038/s41598-019-38968-y (PMC6389966; doi:10.1038/s41598-019-38968-y)
Supplement: Supplementary file 1 — Supplementary Information [file 41598_2019_38968_MOESM1_ESM.docx]

Supplementary information for

Hypochlorous-Acid-Generating Electrochemical Scaffold for

Treatment of Wound Biofilms

Mia Mae Kiamco^1^, Hannah Zmuda^1^, Abdelrhman Mohamed^1^, Douglas R. Call^2^,

Yash S. Raval^3^, Robin Patel^3,4^, and Haluk Beyenal^1,*^

^1^The Gene and Voiland School of Chemical Engineering and Bioengineering and ^2^the Paul G. Allen School for Global Animal Health, Washington State University, Pullman, WA; Divisions of ^3^Clinical Microbiology and ^4^Infectious Diseases, Mayo Clinic, Rochester, MN


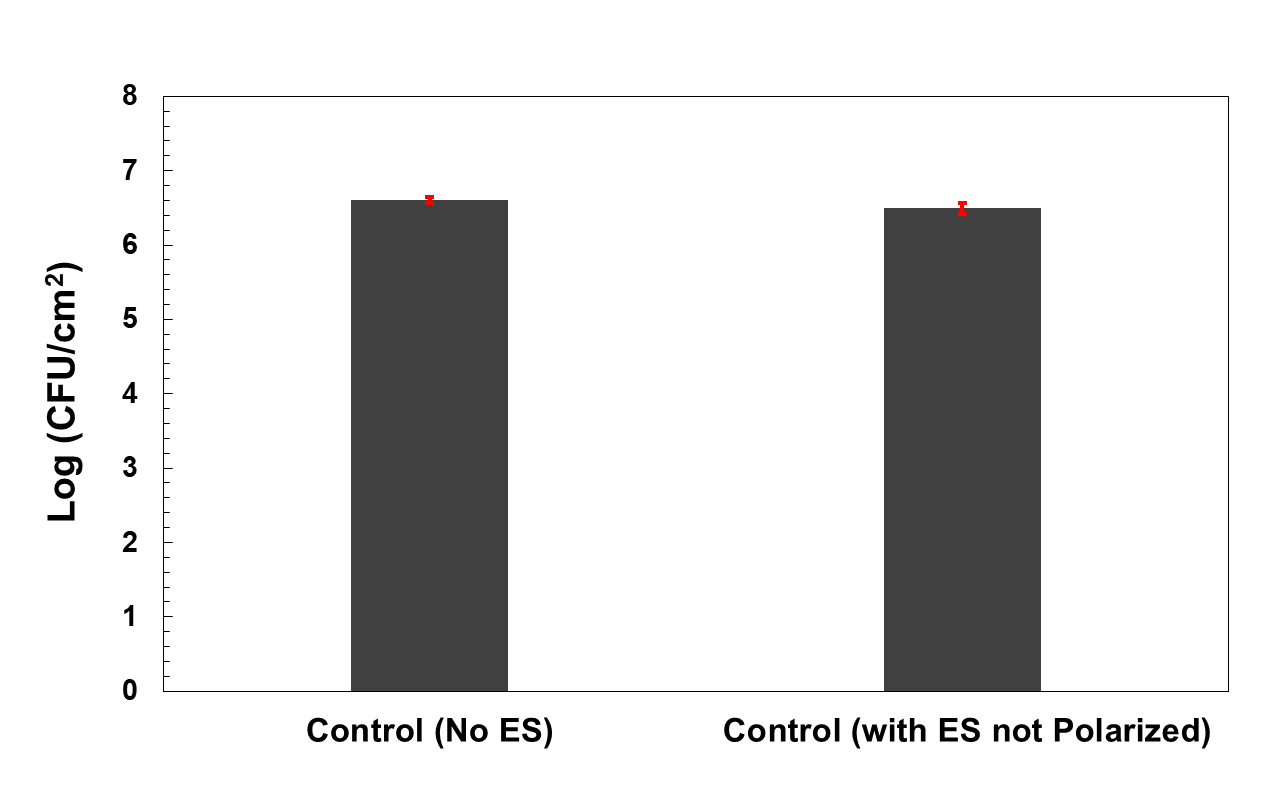


**Figure SI 1.** Controls with no e-scaffold and with non-polarized e-scaffold showing almost identical CFU, indicating that the e-scaffold (with a reference and a counter electrode) by itself does not eradicate *Staphylococcus aureus* biofilms. The e-scaffold was conditioned as described in the main manuscript**.**

**Figure SI 2.** Example calibration curve for HOCl microelectrode.
